# Optical Properties of ZnO Nanoparticles Capped with Polymers

**DOI:** 10.3390/ma4061132

**Published:** 2011-06-17

**Authors:** Shingo Tachikawa, Atsushi Noguchi, Takeharu Tsuge, Masahiko Hara, Osamu Odawara, Hiroyuki Wada

**Affiliations:** Tokyo Institute of Technology, 4259 Nagatsuta-cho, Midori-ku, Yokohama 226-8503, Japan; E-Mails: tachikawa21012@gmail.com (S.T.); longchampgntu04@hotmail.com (A.N.); ttsuge@iem.titech.ac.jp (T.T.); masahara@echem.titech.ac.jp (M.H.); odawara.o.aa@m.titech.ac.jp (O.O.)

**Keywords:** nanoparticle, fluorescence, capping, zinc oxide, polyethylene glycol, polyvinyl pyrrolidone

## Abstract

Optical properties of ZnO nanoparticles capped with polymers were investigated. Polyethylene glycol (PEG) and polyvinyl pyrrolidone (PVP) were used as capping reagents. ZnO nanoparticles were synthesized by the sol-gel method. Fluorescence and absorption spectra were measured. When we varied the timing of the addition of the polymer to the ZnO nanoparticle solution, the optical properties were drastically changed. When PEG was added to the solution before the synthesis of ZnO nanoparticles, the fluorescence intensity increased. At the same time, the total particle size increased, which indicated that PEG molecules had capped the ZnO nanoparticles. The capping led to surface passivation, which increased fluorescence intensity. However, when PEG was added to the solution after the synthesis of ZnO nanoparticles, the fluorescence and particle size did not change. When PVP was added to the solution before the synthesis of ZnO nanoparticles, aggregation of nanoparticles occurred. When PVP was added to the solution after the synthesis of ZnO nanoparticles, fluorescence and particle size increased. This improvement of optical properties is advantageous to the practical usage of ZnO nanoparticles, such as bioimaging.

## 1. Introduction

Nanoparticles have indicated unique properties in materials, which have been different from bulk materials by a reduction in volume and an increase in the specific surface area [1,2,3,4]. Properties, such as band gap engineering of nanoparticles by quantum confinement and increases in reactivity from increased specific surface area, have been investigated by researchers in various fields [5,6,7]. These materials could be utilized for light-emitting devices, fluorescent materials, catalysts and paint. Semiconductor nanoparticles are expected to have uses in bioimaging, displays and fluorescent lights because of the controllability of both the excitation and the emission wavelengths by a quantum size effect. In these applications, bioimaging has been studied widely because of the potential realization of personalized medicine, genomic drug discovery and preventive medicine [8,9]. The research of cadmium series quantum dots, which show visible fluorescence, has led to the development of a nanosize marker for bioimaging. However, the toxicity of cadmium to the human body has been a concern [10,11].

ZnO has been one of the most promising materials for electrical devices, including transparent conductive films, light emitting diodes and photocatalyst [12,13,14,15,16,17]. Moreover, because it has been chemically and optically stable and has a low toxicity, its use as a fluorescent label for bioimaging has been anticipated. When using nanoparticles for biomedical purposes, the prevention of the aggregation of nanoparticles and the nonselective adsorption of the protein has been a serious concern. Capping nanoparticles has been one of the most important methods used to solve these problems. The fluorescence of ZnO has been observed in the UV region due to the exciton luminescence of the band gap and in the visible region due to oxygen defects and/or interstitial zinc caused by UV excitation [12,18,19,20,21,22,23]. Studies of the capping of ZnO nanoparticles by polyvinyl pyrrolidone (PVP) or polyvinyl butyral (PVB) have been previously performed [24,25]. The visible fluorescence intensity of ZnO capped by these polymers was decreased, which was not suitable for bioimaging. Capping by the other polymers which improve the optical properties was necessary for bioimaging. We have observed an increase in fluorescence intensity of ZnO nanoparticles by capping with polyethylene glycol (PEG) [26].

In this study, the influence of polymer capping on the photoluminescence (PL) intensity of ZnO nanoparticles was investigated. In particular, the type of polymer and the timing of the addition of the polymer were examined as critical parameters. The advantages of capping nanoparticles by polymer molecules were the following: (1) the passivation of surface defects, which decreased the nonradiative recombination center, such as dangling bonds, and increased fluorescence intensity [27,28,29]; (2) the aggregation inhibition of nanoparticles by a steric effect; 3) the utilization of nanoparticles for a drug delivery system (DDS) by the improvement in immunoaffinity; and 4) the probability of surface functionalization of nanoparticle, such as targeting.

## 2. Results and Discussion

**Figure 1:**
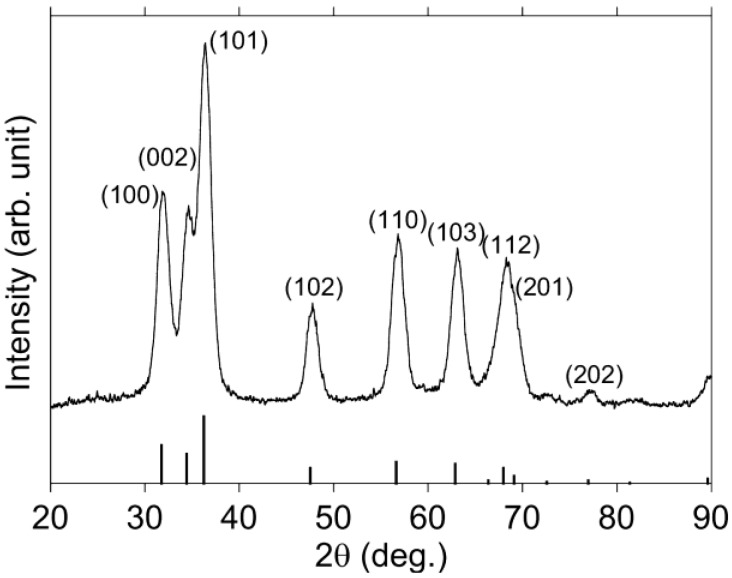
XRD pattern of the synthesized nanoparticles.

The XRD pattern of synthesized nanoparticles is shown in Figure 1. These peaks almost correspond to the PDF data of ZnO (PDF 36-1451, bottom part of Figure 1), which indicated the hexagonal wurtzite structure, although the peaks were broad. Full width at half maximum (FWHM) *B* is inversely proportional to crystallite size *D*, as indicated in the following Scherrer’s Equation (1):

(1) $$B=\frac{K\lambda }{D\text{cos}\theta_{B}}$$

where *K* is the Scherrer constant, *λ* is the wavelength of the X-ray, *θ*_B_ is the Bragg angle [30]. Estimated average particle size was approximately 5 nm.

**Figure 2:**
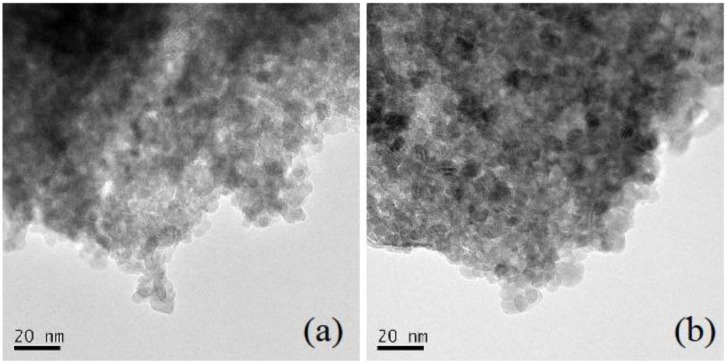
TEM image of the ZnO nanoparticles: (**a**) ZnO nanoparticles without PEG (PEG/Zn^2+^ = 0.0[mol/mol]); (**b**) ZnO nanoparticles with PEG (PEG/Zn^2+^ = 1.2[mol/mol]).

Figure 2a shows TEM image of ZnO nanoparticles without PEG, while Figure 2b shows that of ZnO nanoparticles with PEG. ZnO nanoparticles were washed by hexane after synthesis in order to remove PEG molecules capping ZnO. Particle size without PEG was almost the same as that with PEG. This result corresponds with the following result of absorption spectra. Lattice fringes, which indicated high crystallinity, were clearly observed on each particle. In the case of ZnO, oxide nanoparticles could be easily obtained from the metal salt by the sol-gel method without calcination. The average particle size in the TEM images was almost consistent with the size estimated by the FWHM of the X-ray.

The type of polymer and the timing of the addition of the polymer were varied in order to investigate the influence of capping by the polymer on the optical properties of the ZnO nanoparticles. PEG and PVP were used as the capping polymers. The addition of the polymer to the ZnO solution before the synthesis of ZnO was described as “before synthesis,” while the addition of the polymer to the solution after the synthesis was described as “after synthesis.”

**Figure 3:**
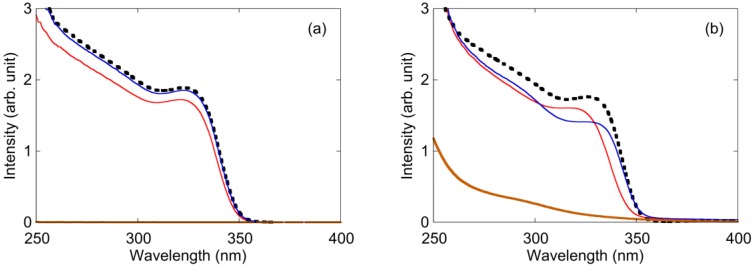
Absorption spectra of the ZnO nanoparticle solution. (**a**) PEG was added to the solution; (**b**) PVP was added to the solution. Black dotted line: polymer was not added to the solution. Red solid line: polymer was added before the synthesis of ZnO nanoparticles. Blue solid line: polymer was added after synthesis. Brown solid line: data of only polymer solution.

To understand the size of the ZnO nanoparticles and its concentration in the solution, absorption spectra were measured. The band-edge of the bulk ZnO material was 3.2 eV (388 nm) at room temperature [31]. However, the band-edge of the ZnO nanoparticle was blue-shifted by the quantum confinement effect when compared with bulk ZnO. The relationship between the bandgap of nanoparticle *E* and particle radius *r* is given by:

(2) $$E≅E^{bulk}+\frac{\hbar^{2}\pi^{2}}{2er^{2}}\left(\frac{1}{m_{e}m_{0}}+\frac{1}{m_{h}m_{0}}\right)-\frac{1.8e}{4\pi \varepsilon \varepsilon_{0}r}$$

where *E*^bulk^ is bulk bandgap, ℏ is Planck’s constant divided by 2*π*, *e* is elementary electric charge, *m*_e_ is the electron effective mass, *m*_h_ is hole effective mass, *m*_0_ is electron mass, ε is relative permittivity, and *ε*_0_ is permittivity of vacuum [31,32,33]. Absorbance was proportional to the concentration of the solution, as described by the Lambert-Beer law. High absorbance meant a high concentration of ZnO nanoparticles. Edge onset and height of the absorption spectrum indicated the size and concentration of the ZnO nanoparticles, respectively. Figure 3 shows the absorption spectra of the ZnO solution and polymer. In the case of PEG (Figure 3a), the absorption edge and height decreased slightly when compared with that of no addition of PEG when PEG was added to the ZnO solution before synthesis. However, absorption spectrum did not change when PEG was added to ZnO solution after synthesis. Particle sizes of “no addition of PEG,” “before synthesis” and “after synthesis” estimated by the absorption edges were 3.7 nm, 3.8 nm and 3.7 nm, respectively. Changes in the concentrations of “before synthesis” and “after synthesis” when compared with “no addition of PEG” were −9% and ±0%, respectively. No absorbance of PEG was observed in this region. In the case of PVP addition before synthesis (Figure 3b), the absorption edge and height decreased slightly. In the case of PVP addition after synthesis, absorption height decreased, although the absorption edge did not change. Particle sizes of “no addition of PVP,” “before synthesis” and “after synthesis” estimated by the absorption edges were 3.8 nm, 3.6 nm and 3.8 nm, respectively. The changes in concentrations of “before synthesis” and “after synthesis” when compared with “no addition of PVP” were −9% and −20%, respectively. Absorbance of PVP was plotted in Figure 5b. The values for the blue-shift in the absorption spectra in Figure 5 indicated that particle sizes of the ZnO nanoparticles were almost the same, even when polymers were added. The concentrations of some solutions were probably decreased by the precipitation of ZnO nanoparticles by aggregation.

**Figure 4:**
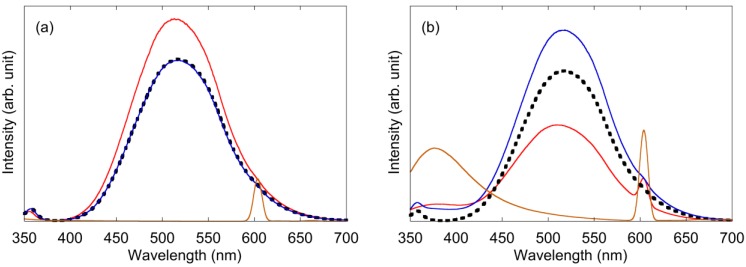
PL spectra of the ZnO nanoparticle solution with an excitation wavelength of 300 nm: (**a**) PEG was added to the solution; (**b**) PVP was added to the solution. Black dotted line: polymer was not added to the solution. Red solid line: polymer was added before the synthesis of ZnO nanoparticles. Blue solid line: polymer was added after the synthesis. Brown solid line: data of only the polymer solution.

Figure 4 shows the PL spectra of the ZnO nanoparticle solution with an excitation wavelength of 300 nm. In general, broad green fluorescence of ZnO was attributed to oxygen defects, which existed both on the inside and on the surface of the ZnO particles [12,18,19,20,21,22,23]. Surface defects, which existed only on the surface of fluorescent material, decreased fluorescence intensity because the surface defect level increased the nonradiative transitions [27,28,29]. Therefore, the fluorescence intensity of the nanoparticles, which had large specific surface areas, was significantly weaker than that of the bulk material. In the case of PEG (Figure 4a), the fluorescence intensity of the “before synthesis” solution (red solid line) increased by 25% when compared to that with no addition of PEG (black dotted line) although absorption, which indicated quantity of ZnO nanoparticles, was decreased. This phenomenon suggested the increase in fluorescent efficiency. In general, capping fluorescent nanoparticles by polymers increased the fluorescence intensity because of passivation of the surface killer defects [27,28,29]. The fluorescence intensity of the PEG addition after synthesis (blue solid line) was exactly the same as that with no addition of PEG. Unlike the results from the addition of PEG before synthesis, passivation by PEG capping did not occur with the addition of PEG after synthesis. PEG should be added to the ZnO solution before the synthesis of the nanoparticles to increase the green fluorescence of ZnO. In the case of PVP (Figure 4b), fluorescence intensity added before synthesis (red solid line) decreased by 36% when compared to that with no addition of PVP (black dotted line). The decrease in green fluorescence by polymer capping was consistent with reports of previous work [24,25], although the UV emission was weak because annealing was not performed after the synthesis of ZnO nanoparticles. However, when PVP was added to the ZnO solution after synthesis, the fluorescence intensity (blue solid line) increased by 27% when compared to that with no addition of PVP (black dotted line). Unlike the results from PEG capping, it was found that PVP should be added to ZnO solution after the synthesis of the nanoparticles to increase the green fluorescence of ZnO. Background between 350 and 420 nm was slightly high because the fluorescence of PVP molecules was observed at 370 nm (brown solid line). A weak peak at 600 nm was attributed to the second order scattering at the grating.

**Figure 5:**
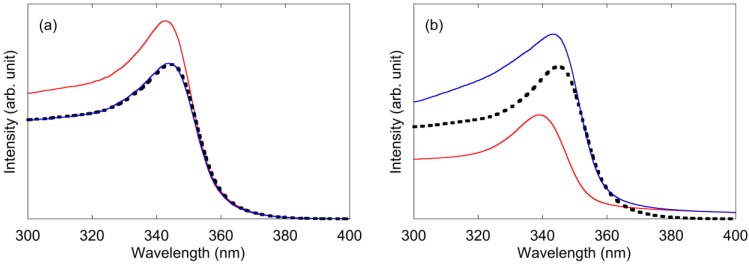
PLE spectra of ZnO nanoparticle solution with an emission wavelength of 500 nm: (**a**) PEG was added to the solution; (**b**) PVP was added to the solution. Black dotted line: polymer was not added to the solution. Red solid line: polymer was added before the synthesis of ZnO nanoparticles. Blue solid line: polymer was added after the synthesis of ZnO nanoparticles.

Figure 5 shows the PLE spectra of ZnO with an emission wavelength of 500 nm. The trend in Figure 5 corresponded to the results of the PL spectra in Figure 4. The peak around 340 nm could be attributed to the bandgap of ZnO nanoparticles, which was blue-shifted by the quantum confinement effect. In the case of PEG addition (Figure 5a), fluorescence of the ZnO nanoparticles increased when PEG was added to the ZnO solution before synthesis. However, the fluorescence did not change when PEG was added to ZnO solution after synthesis. In the case of PVP addition (Figure 5b), fluorescence decreased when PVP was added to the solution before synthesis. Fluorescence increased when PVP was added after synthesis.

**Figure 6:**
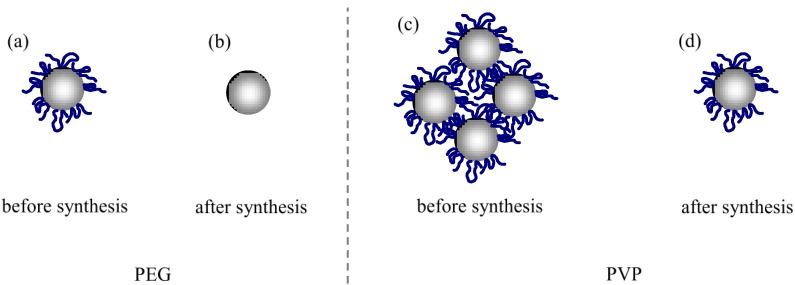
Schematic of ZnO nanoparticles capped by polymer molecules: (**a**) PEG was added to the ZnO solution before synthesis; (**b**) PEG was added to the ZnO solution after synthesis; (**c**) PVP was added to the ZnO solution before synthesis; (**d**) PVP was added to the ZnO solution after synthesis.

In the case of the absorption measurement, the band-edge of ZnO nanoparticle is utilized. Therefore, the particle size of ZnO would be measured [26]. In the case of the dynamic light scattering (DLS) measurement, the time constant of Brownian motion of total particle is utilized. Therefore, the total particle size which included ZnO and polymers would be measured [26]. The thickness of capping polymer would be estimated by the difference between these particle sizes. The change in total particle size of both ZnO and capping polymers estimated by DLS data is shown in Table 1 with the above data of change in the fluorescence intensities and the ZnO particle sizes. The values with addition of polymer were compared to the value without addition of polymer. The data from “before synthesis” and “after synthesis” were compared with that of no addition of polymers. The particle sizes of the ZnO nanoparticle, measured by absorption edge, were almost the same in all experimental conditions. However, the total particle sizes estimated by DLS increased in some experimental conditions. Therefore, it was believed the total particle size increased because the ZnO nanoparticles were capped by the polymer when the polymer was added to the solution. In the case of PEG addition before synthesis, the particle size and fluorescence intensity increased. Nanoparticles have large specific surface areas when compared with the bulk material. There were a number of surface defects on the surface of the nanoparticles, which decreased the fluorescence intensity by nonradiative transitions. In general, the surface passivation of a nanoparticle by polymer-capping increased the fluorescence intensity [27,28,29]. Figure 6a shows the schematic of a capped ZnO nanoparticle, when PEG was added before synthesis, since almost the same particle size of ZnO, and the increase in total particle size suggested the capping of nanoparticle with polymers. However, in the case of PEG addition after synthesis, the capping of nanoparticle with polymers did not happen as shown in Figure 6b, since no change in both ZnO and total particle size was observed. In this condition fluorescence intensity was not observed. In the case of PEG addition, the reason for the discrepancy between the before and after synthesis could be related to the polymer molecules being adsorbed to the ZnO nanoparticles. If the polymer molecules existed in the solution during the synthesis of ZnO nanoparticles, the chance of electrostatic adsorption to ZnO nanoparticles increased. Therefore, PEG molecules probably capped ZnO nanoparticles only when PEG was added to the solution before the synthesis of ZnO nanoparticle. In the case of PVP addition before synthesis, the total particle size increased drastically, although ZnO particle size was almost the same. This change suggested the aggregation of PVP-capped ZnO nanoparticles, as shown in Figure 6c. In this condition, the fluorescence intensity decreased. This decrease in green fluorescence intensity by addition of PVP was similar to previous studies [24,25]. The PVP molecule has a broad fluorescence at 370 nm (Figure 3b). The aggregation of PVP-capped nanoparticles could lead to the increase of UV luminescence of PVP and the decrease of green luminescence by nonradiative transitions, which was similar to concentration quenching. In the case of PVP addition after synthesis, particle size increased, although ZnO particle size was almost the same. This change suggested ZnO nanoparticle was capped with polymers as shown in Figure 6d. In this condition, fluorescence intensity increased, which was similar to the case of PEG addition before synthesis. The discrepancy in the capping behavior between PEG and PVP could be attributed to the difference in adhesion strength. Because PVP has been widely known as a capping reagent for nanoparticles, adhesion of PVP was stronger than that of PEG. During the synthesis of ZnO nanoparticle, pH value of the solution was 6 at the beginning of synthesis and it was approximately 8 at the end of synthesis because of dropping sodium hydroxide. ZnO nanoparticles were positively-charged at the beginning of ZnO synthesis because pH value was less than a point of zero charge of ZnO. In the case of “before synthesis,” polymer was added to the solution in this condition. On the other hand, ZnO nanoparticles were almost not charged at the end of ZnO synthesis because pH value was nearly equal to a point of zero charge of ZnO. In the case of “after synthesis,” polymer was added to the solution in this condition. Although both PEG and PVP are non-ionic surfactants, they would be partially-charged. Especially oxygen in PVP would be charged more strongly than oxygen in PEG because of positively-charged nitrogen in PVP. Therefore, PVP would electrostatically-adhere to the surface of ZnO nanoparticle more strongly than PEG, especially in the case of “before synthesis”. In the case of PVP, the result that the total particle size of the “before synthesis” was larger than “after synthesis” was obtained, which was the same as PEG. In the case of PEG addition before synthesis and PVP addition after synthesis, one of most meaningful results was the increase in fluorescent intensity in spite of having almost the same absorbance as the ZnO nanoparticles. This phenomenon indicated that fluorescent efficiency of ZnO nanoparticle was increased by capping the ZnO nanoparticles.

**Table 1:** Change in size and PL intensity of capped ZnO nanoparticle (The values with addition of polymer were compared to the value without addition of polymer).

		Δ particle size of ZnO (%)	Δ total particle size of ZnO and polymer (%)	Δ PL intensity (%)
PEG	before synthesis	−3	+15	+25
	after synthesis	±0	±0	±0
PVP	before synthesis	−5	+175	−36
	after synthesis	±0	+26	+27

## 3. Experimental Section

Zinc acetate dihydrate, sodium hydroxide, polyethylene glycol, polyvinyl pyrrolidone, ethanol (99.5%) and hexane (Kanto Chemical) were used without further purification. Polymer was added to the solution in which ZnO nanoparticles were dispersed. In this experiment, PEG and PVP were used as the polymers. The polymer addition occurred either “before synthesis” or “after synthesis.” In the case of “before synthesis,” the polymer was added to the solution before the synthesis of the ZnO nanoparticles. In the case of “after synthesis,” the polymer was added to the solution after synthesis of the ZnO nanoparticles. For the “before synthesis” procedure, the addition of the polymer was included before the synthesis of the ZnO nanoparticles by the normal sol-gel method in ethanol [34,35,36,37,38,39]. Zinc acetate dihydrate (1 mmol) and the polymer were ultrasonically dissolved in 50 mL of ethanol at room temperature. Sodium hydroxide (1 mmol) was also ultrasonically dissolved in 50 mL of ethanol in another beaker at room temperature. The solution with sodium hydroxide was dropped into the solution with zinc acetate and the polymer by using a separating funnel, which was stirred vigorously in an Erlenmeyer flask at room temperature. Aging time was 24 hours. Fifty milliliters of ethanol were added to this ZnO solution to make a concentration equivalent to that of the “after synthesis” solution. The “after synthesis” procedure involved the synthesis of ZnO nanoparticles by the sol-gel method in ethanol, described above, without the addition of the polymer. The polymer was ultrasonically dissolved in 50 mL of ethanol in another beaker. After the synthesis of the ZnO nanoparticles, the solution with the polymer was added to the solution with the ZnO nanoparticles.

The powder of nanoparticles was characterized using both X-ray diffraction (XRD) and field-emission transmission electron microscopy (FE-TEM) measurements, which followed the procedures outlined below. A non-polar solvent, hexane, was added to the nanoparticle solution to precipitate the hydrophilic nanoparticle in ethanol by the effect of aggregation of nanoparticles. The ratio of ethanol to hexane was 1:2. Three hours after the addition of hexane, centrifugal separation was performed to obtain the powder of nanoparticles as sediment. The XRD pattern was measured by an X-ray diffractometer (RIGAKU, RINT2000). The size and morphology of the nanoparticle were observed with a FE-TEM (JEOL, JEM-2010F) with an accelerating voltage of 200 kV. The powder of nanoparticles was suspended in ethanol and was dropped on a carbon membrane on a copper grid. The solvent was removed by drying in a vacuum oven. PL spectra and photoluminescence excitation (PLE) spectra of the ZnO nanoparticle in carbon tetrachloride were measured at room temperature with a fluorescence spectrophotometer (Hitachi High-Technologies, F-7000). Absorption spectra were measured at room temperature with a spectrometer (Shimadzu, Multispec1500). The thickness of cell used for absorption, PL, PLE spectra and DLS was 10 mm. The total particle size, including the ZnO nanoparticle and capping polymer, was estimated using DLS (Spectris, Zetasizernano ZS), while the particle size of ZnO nanoparticle was estimated by absorption spectra. The thickness of capping polymer would be estimated by the difference between these particle sizes.

## 4. Conclusions

In the case of PEG addition before synthesis and PVP addition after synthesis, the fluorescent intensity of the ZnO nanoparticles increased, in spite of having almost the same absorbance as the ZnO nanoparticles. The phenomenon indicated that the fluorescent efficiency of the ZnO nanoparticles was increased by capping the ZnO nanoparticles. The addition of these polymers probably led to capping ZnO nanoparticles, which passivated surface defects. This phenomenon depended on the timing of the addition of the polymer to the ZnO solution. The experimental results of the PVP addition before the synthesis of ZnO nanoparticles indicated the aggregation of capped nanoparticles and decreased the fluorescent intensity of the ZnO nanoparticles.

## References

[1] Nirmal M., Brus L. (1999). Luminescence photophysics in semiconductor nanocrystals. Accounts Chem. Res..

[2] Weller H. (1993). Colloidal semiconductor Q-particles: Chemistry in the transition region between solid state and molecules. Angew. Chem. Int. Edit..

[3] Hines M.A., Guyot-Sionnest P. (1996). Synthesis and characterization of strongly luminescing ZnS-capped CdSe nanocrystals. J. Phys. Chem..

[4] Dabbousi B.O., Rodriguez-Viejo J., Mikulec F.V., Heine J.R., Mattoussi H., Ober R., Jensen K.F., Bawendi M.G. (1997). (CdSe)ZnS core-shell quantum dots: Synthesis and characterization of a size series of highly luminescent nanocrystallites. J. Phys. Chem. B.

[5] Efros A.L., Efros A.L. (1982). Interband absorption of light in a semiconductor sphere. Sov. Phys. Semicond..

[6] Bawendi M.G., Steigerwald M.L., Brus L.E. (1990). The quantum mechanics of larger semiconductor clusters (“quantum dots”). Annu. Rev. Phys. Chem..

[7] Alivisatos A.P. (1996). Semiconductor clusters, nanocrystals, and quantum dots. Science.

[8] Bruchez M., Moronne M., Gin P., Weiss S., Alivisatos A.P. (1998). Semiconductor nanocrystals as fluorescent biological labels. Science.

[9] Chan W.C.W., Nie S. (1998). Quantum dot bioconjugates for ultrasensitive nonisotopic detection. Science.

[10] Dubertret B., Skourides P., Norris D.J., Noireaux V., Brivanlou A.H., Libchaber A. (2002). *In vivo* imaging of quantum dots encapsulated in phospholipid micelles. Science.

[11] Derfus A.M., Chan W.C.W., Bhatia S.N. (2004). Probing the cytotoxicity of semiconductor quantum dots. Nano Lett..

[12] Vanheusden K., Seager C.H., Warren W.L., Tallant D.R., Voigt J.A. (1996). Correlation between photoluminescence and oxygen vacancies in ZnO phosphors. Appl. Phys. Lett..

[13] Dayan N.J., Sainkar S.R., Karekar R.N., Aiyer R.C. (1998). Formulation and characterization of ZnO: Sb thick-film gas sensors. Thin Solid Films.

[14] Chen C.S., Kuo C.T., Wu T.B., Lin I.N. (1997). Microstructures and electrical properties of V_2_O_5_-based multicomponent ZnO varistors prepared by microwave sintering process. Jpn. J. Appl. Phys. Part 1.

[15] Gorla C.R., Emanetoglu N.W., Liang S., Mayo W.E., Lu Y., Wraback M., Shen H. (1999). Structural, optical, and surface acoustic wave properties of epitaxial ZnO films grown on (0112) sapphire by metalorganic chemical vapor deposition. J. Appl. Phys..

[16] Tang Z.K., Wong G.K.L., Yu P., Kawasaki M., Ohtomo A., Koinuma H., Segawa Y. (1998). Room-temperature ultraviolet laser emission from self-assembled ZnO microcrystallite thin films. Appl. Phys. Lett..

[17] Reynolds D.C., Look D.C., Jogai B. (1996). Optically pumped ultraviolet lasing from ZnO. Solid State Commun..

[18] Liu M., Kitai A.H., Mascher P. (1992). Point-defects and luminescence-centers in zinc-oxide and zinc-oxide doped with manganese. J. Lumin..

[19] Vanheusden K., Warren W.L., Seager C.H., Tallant D.R., Voigt J.A., Gnade B.E. (1996). Mechanisms behind green photoluminescence in ZnO phosphor powders. J. Appl. Phys..

[20] Li D., Leung Y.H., Djurisic A.B., Liu Z.T., Xie M.H., Shi S.L., Xu S.J., Chan W.K. (2004). Different origins of visible luminescence in ZnO nanostructures fabricated by the chemical and evaporation methods. Appl. Phys. Lett..

[21] Djurisic A.B., Choy W.C.H., Roy V.A.L., Leung Y.H., Kwong C.Y., Cheah K.W., Rao T.K.G., Chan W.K., Lui H.T., Surya C. (2004). Photoluminescence and electron paramagnetic resonance of ZnO tetrapod structure. Adv. Funct. Mater..

[22] Garces N.Y., Giles N.C., Halliburton L.E., Cantwell G., Eason D.B., Reynolds D.C., Look D.C. (2002). Production of nitrogen acceptors in ZnO by thermal annealing. Appl. Phys. Lett..

[23] Xu P.S., Sun Y.M., Shi C.S., Xu F.Q., Pan H.B. (2003). The electronic structure and spectral properties of ZnO and its defects. Nucl. Instrum. Methods Phys. Res..

[24] Guo L., Yang S.H., Yang C.L., Yu P., Wang J.N., Ge W.K., Wong G.K.L. (2000). Highly monodisperse polymer-capped ZnO nanoparticles: Preparation and optical properties. Appl. Phys. Lett..

[25] Guo L., Yang S.H., Yang C.L., Yu P., Wang J.N., Ge W.K., Wong G.K.L. (2000). Synthesis and characterization of poly(vinylpyrrolidone)-modified zinc oxide nanoparticles. Chem. Mater..

[26] Tachikawa S., Noguchi A., Hara M., Odawara O., Wada H. (2011). Structures and optical properties of ZnO nanoparticles capped with polyethylene glycol. J. Ceram. Process. Res..

[27] Pankove J.I. (1971). Optical Processes in Semiconductors.

[28] Micic O.I., Sprague J.R., Lu Z., Nozik A. (1996). Highly efficient band-edge emission from InP quantum dots. Appl. Phys. Lett..

[29] Kuno M., Lee J.K., Dabbousi B.O., Mikulec F.V., Bawendi M.G. (1997). The band edge luminescence of surface modified CdSe nanocrystallites: Probing the luminescing state. J. Chem. Phys..

[30] Cullity B.D. (1978). Elements of X-ray Diffraction.

[31] Pesika N.S., Stebe K.J., Searson P.C. (2003). Determination of the particle size distribution of quantum nanocrystals from absorbance spectra. Adv. Mater..

[32] Hu Z., Oskam G., Searson P.C. (2003). Influence of solvent on the growth of ZnO nanoparticles. J. Collo. Inter. Sci..

[33] Brus L.E. (1986). Electronic wave-functions in semiconductor clusters—Experiment and theory. J. Phys. Chem..

[34] Koch U., Fojtik A., Weller H., Henglein A. (1985). Preparation of extremely small ZnO particles, fluorescence phenomena and size quantization effects. Chem. Phys. Lett..

[35] Bahnemann D.W., Kormann C., Hoffmann M.R. (1987). Preparation and characterization of quantum size zinc-oxide—a detailed spectroscopic study. J. Phys. Chem..

[36] Haase M., Weller H., Henglein A. (1988). Electron storage on ZnO particles and size quantization. J. Phys. Chem..

[37] Spanhel L., Anderson M.A. (1991). Semiconductor clusters in the sol-gel process—Quantized aggregation, gelation, and crystal-growth in concentrated ZnO colloids. J. Am. Chem. Soc..

[38] Meulenkamp E.A. (1998). Synthesis and growth of ZnO nanoparticles. J. Phys. Chem. B.

[39] Spanhel L. (2006). Colloidal ZnO nanostructures and functional coatings: A survey. J. Sol-Gel Sci. Technol..

